# Methods for Lipid Droplet Biophysical Characterization in *Flaviviridae* Infections

**DOI:** 10.3389/fmicb.2018.01951

**Published:** 2018-08-21

**Authors:** Ana S. Martins, Ivo C. Martins, Nuno C. Santos

**Affiliations:** Instituto de Medicina Molecular, Faculdade de Medicina, Universidade de Lisboa, Lisbon, Portugal

**Keywords:** lipid droplet, *Flaviviridae*, viral proteins, LDs-associated proteins, light scattering, microscopy

## Abstract

Lipid droplets (LDs) are intracellular organelles for neutral lipid storage, originated from the endoplasmic reticulum. They play an essential role in lipid metabolism and cellular homeostasis. In fact, LDs are complex organelles, involved in many more cellular processes than those initially proposed. They have been extensively studied in the context of LD-associated pathologies. In particular, LDs have emerged as critical for virus replication and assembly. Viruses from the *Flaviviridae* family, namely dengue virus (DENV), hepatitis C virus (HCV), West Nile virus (WNV), and Zika virus (ZIKV), interact with LDs to usurp the host lipid metabolism for their own viral replication and pathogenesis. In general, during *Flaviviridae* infections it is observed an increasing number of host intracellular LDs. Several viral proteins interact with LDs during different steps of the viral life cycle. The HCV core protein and DENV capsid protein, extensively interact with LDs to regulate their replication and assembly. Detailed studies of LDs in viral infections may contribute for the development of possible inhibitors of key steps of viral replication. Here, we reviewed different techniques that can be used to characterize LDs isolated from infected or non-infected cells. Microscopy studies have been commonly used to observe LDs accumulation and localization in infected cell cultures. Fluorescent dyes, which may affect LDs directly, are widely used to probe LDs but there are also approaches that do not require the use of fluorescence, namely stimulated Raman scattering, electron and atomic force microscopy-based approaches. These three are powerful techniques to characterize LDs morphology. Raman scattering microscopy allows studying LDs in a single cell. Electron and atomic force microscopies enable a better characterization of LDs in terms of structure and interaction with other organelles. Other biophysical techniques, such as dynamic light scattering and zeta potential are also excellent to characterize LDs in terms of size in a simple and fast way and test possible LDs interaction with viral proteins. These methodologies are reviewed in detail, in the context of viral studies.

## Introduction

**FIGURE 1:**
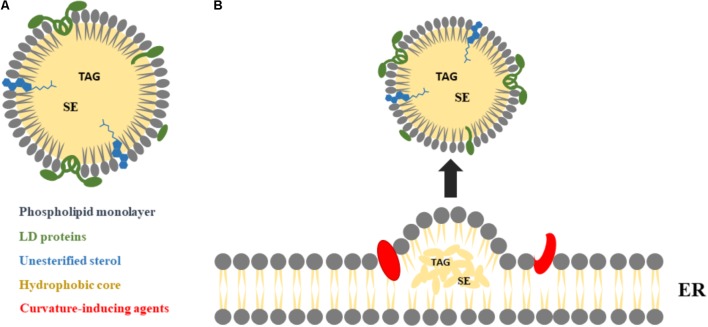
Major LDs morphological features and biogenesis. **(A)** LDs are composed by a neutral core of triacylglycerols (TAGs) and sterol esters (SEs), surrounded by a monolayer of phospholipids and unesterified sterol, with several proteins at the surface and/or partially integrated within their structure, mainly from the PAT family: perilipin 1 (PLIN1), perilipin 2 (PLIN2), and perilipin 3 (PLIN3). **(B)** LDs are derived from the endoplasmic reticulum (ER), as a result of TAG and SE molecules accumulation between the two leaflets of the ER membrane. The nascent droplets grow into mature LDs, with the help of curvature-inducing agents, and may remain attached to the ER (not shown) or detach from the ER into the cytosol.

Lipid droplets (LDs) are intracellular organelles for neutral lipid storage (Walther and Farese, 2012; Hashemi and Goodman, 2015), originated from the endoplasmic reticulum (ER) where some enzymes involved on the generation of neutral lipids are located (Buhman et al., 2001; Hashemi and Goodman, 2015). Mature LDs are composed of a hydrophobic core of neutral lipid, mainly triacylglycerols (TAGs) and sterol esters (SEs), surrounded by a monolayer of phospholipids and unesterified sterol, with a variety of integral and peripheral proteins (Lin P. et al., 2014; Bersuker and Olzmann, 2017). The main LD proteins are from the PAT family: perilipin (also known as perilipin 1, PLIN1), adipose differentiation-related protein (ADRP), also named perilipin 2 (PLIN2), and tail-interacting protein of 47 kDa (TIP47), also named perilipin 3 (PLIN3) (Bickel et al., 2009; Krahmer et al., 2009). LDs tend to have a globular shape (**Figure 1A**) with a diameter that varies from 50 nm to 200 μm, depending on cell type (Krahmer et al., 2009). LDs are the main cell reservoir of lipids for energy production (Krahmer et al., 2009), as well as of sterols, fatty acids, and phospholipids for hormone synthesis and membrane formation (Thiam et al., 2013), minimizing the volume necessary for their storage. Moreover, LDs protect cells from the lipotoxic effects of unesterified lipids (Krahmer et al., 2009). Via their surface, LDs control lipases accessibility to stored TAG, helping to regulate their enzymatic breakdown (Lin P. et al., 2014).

During the last decades, several studies have been carried out to understand LDs biogenesis (Pol et al., 2014; Deslandes et al., 2017; Choudhary et al., 2018) and functions (Walther and Farese, 2012; Welte, 2015). Different model systems, including bacteria, yeast, green algae, *Caenorhabditis elegans*, *Drosophila*, plants and several types of mammalian cells and tissues have been used. Data from several studies show that LDs are very complex organelles and may be involved in lipid metabolism, membrane biosynthesis, membrane trafficking and signal transduction (Ding et al., 2013). LDs were proposed to be involved in many other important processes: LDs modulate nuclear functions being responsible for the availability of proteins and signaling lipids in the nucleus; LDs may act as hubs for fatty acid trafficking to mitochondria; LDs are used by the immune system against pathogens; however, viruses have evolved strategies to use LDs as platforms for viral assembly (Welte, 2015; Wang, 2016).

Lipid droplets have been extensively studied, in particular in the context of LD-associated pathologies. Given the available knowledge about these organelles as regulators of lipid and energy metabolism, their involvement in human metabolic diseases as well as in viral infections is not surprising. In fact, the accumulation of LDs occurs during the progression of different pathologies (Pol et al., 2014). Several important intracellular pathogens from the *Flaviviridae* family, such as hepatitis C virus (HCV) (Barba et al., 1997) and dengue virus (DENV) (Samsa et al., 2009), increase the formation of LDs in the host cells. It has been shown that viral RNA replication is regulated by viral proteins through their interaction with LD surface proteins (Vogt et al., 2013). Viruses of the *Flaviviridae* family cause several serious human conditions, such as hemorrhagic fever (Rigau-Pérez, 2006), liver steatosis (McLauchlan, 2009), and microcephaly (Calvet et al., 2016), caused by DENV, HCV, and Zika virus (ZIKV) infection, respectively.

Here, we review different approaches to characterize LDs in the context of *Flaviviridae* infections, namely DENV and HCV infections. Due to the importance of LDs as key components for viral replication, detailed studies of these organelles and their interaction with viral factors are crucial. This can lead to novel inhibitors of key steps of the viral replication of important human pathogens of the *Flaviviridae* family.

## LDs Biogenesis

Lipid droplets biogenesis starts following the accumulation of TAG and SE molecules between the two leaflets of the ER membrane (Joshi et al., 2017; Thiam and Beller, 2017). To maintain stability, these ER lipid bilayers accommodate neutral lipids, but only up to a saturation point, above which the formation of LDs is triggered (**Figure 1B**) (Hamilton, 1989; Hashemi and Goodman, 2015). Although LDs can be formed spontaneously from the ER (Deslandes et al., 2017), the transition to a mature LD involves structural changes (Vanni, 2017). Proteins may play an essential role on LDs formation (Hashemi and Goodman, 2015) and in the structural changes leading to their maturation (Vanni, 2017). These proteins include PLIN3, as well as proteins containing helical hairpins, such as glycerol-3-phosphate acyltransferase 4 (GPAT4) or diacylglycerol acyltransferase (DGAT) 1 and 2, fat storage-inducing transmembrane protein 2 (FIT2) and seipin (Pol et al., 2014; Hashemi and Goodman, 2015; Choudhary et al., 2018). Molecular dynamics studies suggest that proteins that are recruited to the ER membrane are then expelled precisely at the sites of LDs formation, as a consequence of changes in the underlying membrane properties (Vanni, 2017). Lipids such as diacylglycerol (DAG) and phosphatidic acid also contribute to LDs formation, promoting shape change in the same direction (required for the curvature formation) (Skinner et al., 2009; Adeyo et al., 2011; Hashemi and Goodman, 2015; Choudhary et al., 2018). Moreover, the role of DAG on LDs formation may involve more than its membrane-curvature properties (Hashemi and Goodman, 2015). Nevertheless, the hypothesis that LDs can be spontaneously formed from a symmetrical elongated lens of the ER membrane without requiring any energy-consuming machinery, curvature-inducing agent or intrinsic asymmetry of the bilayer is still a matter of debate (Deslandes et al., 2017). Although extensive studies of LDs biogenesis have been conducted for several years, some questions still remain unanswered. One of them is related with the location of the formation of LDs at the ER. It is not clear if there are specific sites or if it is a process that occurs at random locations (Hashemi and Goodman, 2015; Thiam and Beller, 2017). Another question is if the nascent LDs, as they transit to mature LDs, separate from the ER and migrate from the cell periphery to the nucleus (Hashemi and Goodman, 2015). Answering these questions will contribute to our understanding of cell machinery, helping to clarify the interactions of LDs with other cell organelles.

## LDs in Health and Disease

Lipid droplets form stable associations not only with ER, but also with other key organelles and cellular compartments, like mitochondria, inner nuclear envelope, lysosomes/vacuoles, and endosomes (Welte, 2015; Schuldiner and Bohnert, 2017). Assuming LDs as the source of lipids for other organelles, these contacts may serve to transfer lipids to other compartments, enabling membrane expansion, signaling, and energy production through lipolysis and β-oxidation to occur. For some of these connections, the molecules responsible for initiating or maintaining the contact were already identified, in particular for LDs contact with ER and mitochondria (Gao and Goodman, 2015). Seipin, lipin, and FIT2 are some of the proteins involved in LDs formation, assembly and TAG transfer to LDs, respectively. PLIN5 was identified as a mediator of droplet–mitochondrial interactions, modulating LD lipases (Wilfling et al., 2013; Gao and Goodman, 2015). However, the role of LDs trafficking within the nucleus, as well as the process of energy release from LDs, is still not well understood (Wang, 2016). The neutral lipids stored inside of LDs, such as TAG, SE and retinyl esters, can be used by cells on several biological processes. For instance, LDs are critical for energy and membrane components generation (Welte, 2015). An impairment on LD biogenesis and/or increased LD degradation can disrupt the normal lipid metabolism inside the cell, as well as their energy homeostasis (Greenberg et al., 2011; Krahmer et al., 2013). LDs are important organelles in adipose tissue, liver and intestine, due to their involvement on energy storage and lipid turnover (Bickel et al., 2009; Gross and Silver, 2014). Given their role in lipid storage, LDs also figure prominently in several pathologies due to lipid accumulation, such as obesity, fatty liver, type 2 diabetes and atherosclerosis (Cohen et al., 2011; Greenberg et al., 2011; Walther and Farese, 2012; Krahmer et al., 2013). The increasing number of LDs in non-adipose tissues is a pathological feature of these metabolic diseases (Greenberg et al., 2011; Krahmer et al., 2013). The association of LDs accumulation with these diseases is well understood: LDs are able to sequester toxic lipids, turning them into TAG and storing them, which prevents the lipotoxicity caused by free fatty acids (Bersuker and Olzmann, 2017). Furthermore, it was reported that mutations in proteins directly associated with LDs structure and function may lead to familial lipodystrophies and neutral lipid storage diseases (Greenberg et al., 2011; Krahmer et al., 2013). LDs accumulation also occurs in skeletal muscle, macrophages, mammary glands, adrenal cortex, ovary, and testis (Walther and Farese, 2012). In the last three cases, LDs provide the precursor for the synthesis of cholesterol-driven steroid hormones, including glucocorticoids such as cortisol, mineralocorticoids such as aldosterone, testosterone and estrogens. LDs-associated proteins such as ADRP (or PLIN2) play a significant role in regulating the intracellular distribution of phospholipids and lipids in general. The redistribution of LDs occurs probably due to the reduced number of the LD-surface protein ADRP, responsible for maintaining the dispersed intracellular distribution of these organelles (McIntosh et al., 2010).

## LDs Proteome

Now that the role of LDs in lipid metabolism is better understood, a part of the focus of the most recent research is on other roles that LDs play. Most of these emerging roles that are starting to be studied are associated with particular LD proteins. Thus, understanding LDs proteome and protein targeting are some of the main objectives of recent studies (Goodman, 2018). LDs have been proposed to sequester proteins and, as a result, either modulate their ability to interact with their binding partners or simply store damaged proteins before degradation. They can also promote the assembly of protein complexes (Hodges and Wu, 2010). However, it is not well understood yet if these proteins are stably targeted to the LDs and how the release is controlled.

The notion of LDs as unique organelles in the control of proteins cycle inside cells led to the study of LDs proteome in different types of cells, representative of different organisms or tissues (Goodman, 2018). However, a detailed and accurate examination of the LDs proteome is challenged by the difficulty of obtaining purified LDs completely separated from other associated organelles. One way to tackle this issue is by excluding data in which proteins that are markers of other organelles are identified. However, it is not the best approach, as it is not possible to ascertain that such protein markers are never found in LDs (Bersuker and Olzmann, 2017). Thus, understanding the role of LD-associated proteins requires the accurate definition of LDs proteome (Bersuker et al., 2018). To do so, it is necessary to isolate pure LDs. Several methods for isolating LDs have been established, developed both for proteomic and functional studies, with LDs isolated from different cells. Increasing LDs purity is still one of the major goals, in order to ensure a reproducible amount of high-quality LDs (Ding et al., 2013).

## The *Flaviviridae* Family

The *Flaviviridae* family of viruses is divided into four genera: *Hepacivirus*, *Flavivirus*, *Pestivirus* and the recently proposed *Pegivirus* (Shi et al., 2016). HCV belongs to the first genus, while other important human pathogens such as DENV, yellow fever, West Nile (WNV), ZIKV and tick-borne encephalitis viruses all belong to the genus *Flavivirus* (Mukhopadhyay et al., 2005; Kilpatrick, 2011; Calvet et al., 2016; Shi et al., 2016). The last two genera include animal viruses of less direct relevance for human health. In addition, there are also the so called “flavi-like” viruses, isolated from a range of arthropod species. They are considered distant relatives of the known *Flaviviridae*, which may come to be classified into that taxon (Shi et al., 2016). The better studied *Flaviviridae* are HCV, DENV, WNV, and, recently, ZIKV, among other. In common, these viruses share a single stranded positive sense RNA [ss(+)RNA], with members of the *Hepacivirus* and *Flavivirus* genera having genomes between 9 and 13 kb (Shi et al., 2016).

**FIGURE 2:**
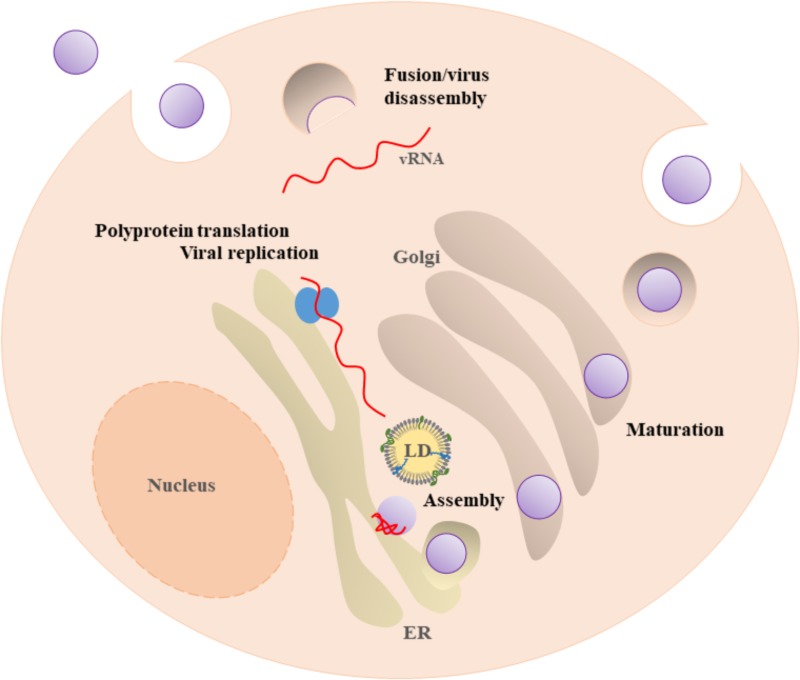
*Flavivirus* life cycle. *Flavivirus* enter in the host cell by receptor-mediated endocytosis. Acidification of the endosomal vesicle leads to the fusion of the viral and cell membranes, enabling the release of the viral genome ss(+)RNA into the cytosol. The ss(+)RNA is translated into a polyprotein that is processed by viral and host proteases, originating seven non-structural and three structural proteins (not shown). Virus replication and assembly occur near the ER and LDs. After virion maturation by the host protease furin, the mature virion follows the secretion pathway and is subsequently released by exocytosis.

Besides their common structure, *Flaviviridae* replication mechanisms are very similar, involving the translation of a single open reading frame into a polyprotein. This polyprotein is cleaved and processed, to later on form the mature virion. The life cycle begins with the attachment of the virus to the cell surface (**Figure 2**). Subsequently, viruses are internalized by receptor-mediated endocytosis and transported to endosomes. Inside the cell, acidification of the endocytic vesicles with viral particles triggers conformational rearrangements in the virion that allows the release of the viral genome into the cytoplasm. The ss(+)RNA is translated as a single polyprotein that it is cleaved by several viral and host proteases, originating non-structural and structural proteins (Mukhopadhyay et al., 2005; Hussmann et al., 2014). Genome replication occurs on intracellular membranes. After the synthesis of viral proteins and of viral RNA, the process of assembly and encapsulation occur on the ER surface. These processes involve several proteins and membrane interactions. The immature viral particles are transported through the trans-Golgi network, where maturation occurs, resulting into infectious particles. The mature infectious particles are then released into the extracellular medium (Mukhopadhyay et al., 2005). A mechanistic understanding of the assembly and encapsulation processes (as well as of other steps of the viral life cycle) may suggest new targets for future therapeutics approaches (Zhang et al., 2017).

## LDs Role in *Flaviviridae* Infections

Positive-sense RNA viruses hijack the intracellular membrane machinery for viral replication (Tang et al., 2014), increasing the number of intracellular LDs and their diameter. LDs are used by viruses as an energy and lipids reservoir (Samsa et al., 2009; Heaton and Randall, 2010; Heaton et al., 2010; Perera et al., 2012). Besides, LDs may also facilitate viral replication, providing a platform for the assembly and encapsidation processes (Samsa et al., 2009). LDs also contribute to viral genome replication. As this process involves an active consumption of cell energy, DENV has been proposed to use the energy stored in LDs through the process of lipophagy (Heaton and Randall, 2010). Briefly, DENV induces autophagy of LDs to release free fatty acids, resulting in an increase of cellular β-oxidation and consequently in an increase of the ATP generated. These processes correlates with the decrease of the LDs area observed in DENV infected cells (Heaton and Randall, 2010).

**FIGURE 3:**
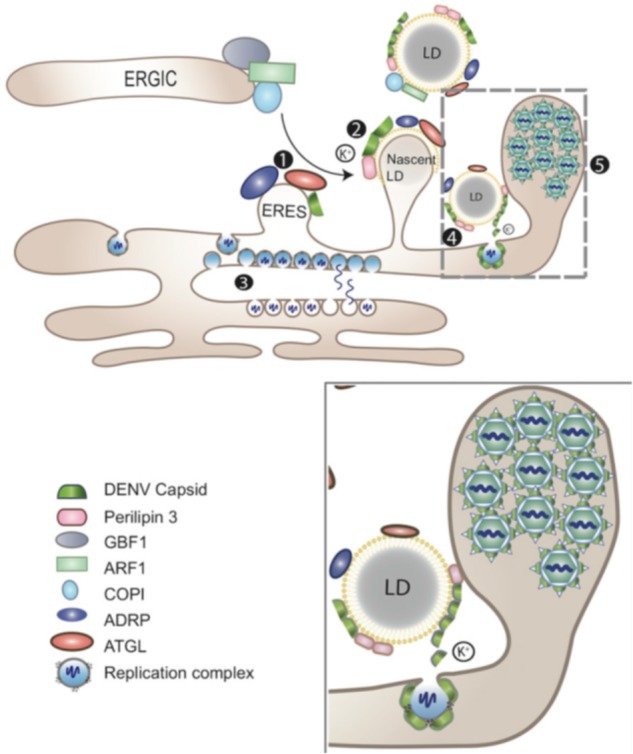
LDs as platform for DENV assembly. LDs (1) at the ER-Golgi intermediate compartment (ERGIC), ARF1 and its guanine nucleotide exchange factor (GEF) GBF1, together with COPI, deliver adipose triacylglycerol lipase (ATGL) and ADRP (perilipin 2) from ER export sites (ERES) to the surface of LDs. DENV subverts this process for the transportation of the C protein to LDs surface. (2) The accumulation of DENV C on LDs depends on perilipin 3 and intracellular K^+^ concentration. (3) Replicated viral genomes are released through the vesicle pore and then engaged into nucleocapsids that bud through the ER membrane in close proximity. (4) DENV C can be released from LDs to the cytosol or other cellular compartments for subsequent viral assembly (gray-dashed frame and enlarged panel). (5) Packed virions accumulate within the lumen of the vesicle packets-containing ER network before being transported to the Golgi (adapted from Zhang et al., 2017).

As mentioned above, *Flaviviridae* and other viruses, such as rotaviruses, use LDs as platforms for viral assembly (**Figure 3**) (Roingeard and Melo, 2017; Zhang et al., 2017). This is achieved through the interaction of LDs with viral proteins, namely the equivalent core and capsid (C) proteins from HCV and DENV, respectively, which play multiple roles during the viral life cycle (Roingeard and Melo, 2017; Zhang et al., 2017). Recently, it was shown that LDs are also targeted by ZIKV C protein (Martins et al., 2017; Shang et al., 2018). Moreover, ZIKV C–LDs interaction can occur in the absence of other viral proteins (Shang et al., 2018). Interestingly, LDs interaction with viral proteins has many more nuances, with LDs proteins playing specific roles.

### HCV Core Protein–LDs Interaction

Lipid droplets play an important role in HCV life cycle and are markers involved in steatosis (Miyanari et al., 2007; McLauchlan, 2009). HCV takes advantage of host intracellular lipid systems, in particular LDs, manipulating their production and distribution inside the cells. In fact, HCV induces an increase in size and number of LDs in host cells (Miller and Krijnse-Locker, 2008; McLauchlan, 2009). Several reports have documented the intracellular localization of HCV core protein associated to LDs, suggesting that this interaction is important in HCV life cycle (Barba et al., 1997; McLauchlan and Hope, 2000). The core protein, a structural protein at the N-terminal of the polyprotein encoded by the viral ss(+)RNA, is a dimeric α-helical protein with two domains (D1 and D2) (McLauchlan, 2000; Boulant et al., 2005, 2006). Studies by several laboratories provided detailed information about this interaction (Boulant et al., 2007). HCV core protein co-localizes with LDs in infected cells in a time-dependent manner. It has been discovered that the association of the core to the LDs is mediated by DGAT1, a host enzyme that synthesizes TAG in the ER (Herker et al., 2010). Briefly, the core protein progressively attaches to the LDs surface, coating it, after which the LDs coated with core protein start to aggregate around the nucleus (Boulant et al., 2008). Most importantly, disrupting the ability of core to coat LDs leads to an inhibition of virus production, which shows the importance of LDs in the viral life cycle (Boulant et al., 2007). The D2 domain of HCV core protein was identified as crucial determinant for its binding to LDs, an interaction essential for viral assembly (Shavinskaya et al., 2007). The interaction of the core protein with LDs is also dependent of a C-terminal domain of the core protein, highly conserved between different viruses. This interaction originates loci, where viral RNA and non-structural proteins involved in genome replication were detected (McLauchlan, 2009). These evidences suggest that these loci may be where the assembly and production of nascent virions occurs. In HCV infection, LDs associate also with microtubules and aggregate mainly around the microtubule-organizing center. The association of the core protein to LDs may also promote their redistribution and accumulation around the nucleus. Such LDs–core protein association may then increase the probability of interactions between the sites of HCV RNA replication and of virion assembly (Boulant et al., 2008).

When associated with the LDs, the HCV core protein interferes with TAG turnover, stabilizing LDs and consequently leading to steatosis (Harris et al., 2011). However, LDs proteins also play an important role in this process. In the absence of PLIN3, HCV core protein-induced steatosis does not occur (Ferguson et al., 2017). Moreover, core association to LDs leads to an inhibition of lipolysis by interfering with the activity of adipose triacylglycerol lipase (ATGL), the enzyme responsible for the first step of degradation of TAG. The core protein alters the binding of ATGL to its activator, comparative gene identification 58, enhancing the association of both proteins with LDs (Camus et al., 2014). Ikβ kinase-α is another critical host factor for HCV-induced lipogenesis (Li et al., 2013). In fact, HCV interacts extensively with host factors to manipulate the lipid metabolism and promote virus assembly, which likely contributes to viral replication and steatosis. LDs are also target by antiviral proteins that compete with viral proteins. Viperin, an interferon-induced antiviral protein, binds to LDs, inhibiting HCV (Hinson and Cresswell, 2009).

### HCV NS5 Protein–LDs Interaction

Hepatitis C virus core protein may be the only protein responsible for intracellular LDs redistribution, but there are other host and viral factors mediating the interaction of the core with LDs, as well as other important processes during viral infection. NS5A, a non-structural protein of HCV, is also a key protein for HCV pathogenesis and persistence. It was previously described that NS5A co-localizes with the core protein (Boulant et al., 2007). The C-terminal domain III of NS5A was identified as determinant for co-localization of the core protein and NS5A at the LDs surface, which is crucial for viral assembly (Appel et al., 2008). The core protein–NS5A interaction on LDs surface is stabilized by apolipoprotein J, also known as clusterin (Lin C.C. et al., 2014). Besides NS5A, other non-structural proteins are involved in HCV assembly. HCV core protein recruits these non-structural proteins and replication complexes to LD-associated membranes, a crucial process for producing new virus particles (Miyanari et al., 2007). HCV NS5 also modulates the function of a K^+^-specific channel (Kv2.1) (Mankouri et al., 2009). Moreover, HCV uses a viroporin, p7, to promote membrane permeability to potassium and other cations in its infection process (Griffin et al., 2003).

### DENV Capsid Protein–LDs Interaction

Dengue virus originates 390 million infections worldwide and in the most severe cases, the disease progresses to dengue hemorrhagic fever (DHF) (Bhatt et al., 2013; Póvoa et al., 2014; Guo et al., 2017). The urgent need of an effective vaccine led to several studies aiming to understand the key steps of the virus life cycle. The viral assembly and encapsidation processes, which are mediated by the C protein and involve LDs, have been studied by us and others in some detail. It is now clear that the mature DENV C protein accumulates on the surface of LDs via an interaction that involves specific hydrophobic amino acids. Briefly, L50 and L54, in the α2 helix of the C protein, were identified as essential for DENV C–LDs binding (Samsa et al., 2009; Martins et al., 2012). The positively charged N-terminal region of the C protein also prompts this interaction (Martins et al., 2012). DENV C interacts mainly with PLIN3, at the LDs’ surface, and this interaction is dependent of the high intracellular concentration of potassium ions (Carvalho et al., 2012). By inhibiting the Na^+^/K^+^-ATPase in DENV-infected cells, without affecting RNA replication, the potassium ions intracellular concentration can be lowered, which in turn prevents the C protein from interacting with LDs and, consequently, decreases the number of viral particles formed (Carvalho et al., 2012). It was proposed that DENV uses a non-canonical function of the COPI system for C protein accumulation on LDs (Iglesias et al., 2015). As with HCV core protein, DENV C–LDs binding is crucial for viral replication (Samsa et al., 2009). Disrupting DENV C association on the LDs surface decreases viral RNA amplification. It also impairs viral particle formation.

### DENV NS4A Protein–LDs Interaction

It was recently proposed that NS4, a non-structural protein of DENV with host immune-response modulation properties, have a key role in the viral life cycle (Gopala Reddy et al., 2018). NS4A is cleaved from NS3 at its N-terminal region and from 2K fragment at its C-terminal. The cleavage of the 2K fragment is essential for NS4A to successfully induce host membrane alterations (Miller et al., 2007). In DENV infection, NS4A associates with a protein localized at LDs and ER: the ancient ubiquitous protein 1 (AUP1) (Zhang et al., 2018). This protein appears predominantly in the mono-ubiquitylated form in non-infected cells. However, in DENV infection it was reported that AUP1 appears in the unmodified form and its expression is enhanced (Zhang et al., 2018). Interestingly, a different AUP1 distribution was observed in DENV-infected cells (Zhang et al., 2018). AUP1 associates to NS4A and relocalizes from LDs to autophagosomes. NS4A interaction with the unmodified AUP1, activates its acyltransferase domain to trigger lipophagy. This process is also dependent of NS4B. Importantly, the ubiquitylation of NS4A disrupts NS4A-AUP1 interaction and, consequently, the lipophagy process essential for flaviviruses infection. Moreover, in the absence of AUP1, cells seem to be resistant to DENV, ZIKV, and WNV production (Zhang et al., 2018).

### Similarities Between HCV, DENV, and Other Flaviviruses

As implied from the above, DENV and HCV have identical interactions between viral and cellular proteins to promote physical contacts with LDs. The Rab18 protein, a member of the Rab GTPase family, is present in LDs and ER membranes, interacting with NS5A and NS3, non-structural proteins of HCV (Salloum et al., 2013) and DENV (Tang et al., 2014), respectively. Rab18 co-localizes with HCV NS5A at LDs surface and seems to promote the physical association of NS5A and LDs, as well as other components for viral replication (Salloum et al., 2013). In DENV infection, Rab18 seems to coordinate the localization of fatty acid synthase (FAS), a key enzyme for lipid biosynthesis, on LDs and ER and its interaction with NS3. Rab18 can be an important host factor to ensure that virus replication occurs at precise locations with sufficient lipid supply (Tang et al., 2014).

## Lipid Droplet Isolation and Purification

The isolation and purification of host lipid systems can be very difficult, if not impossible. Obtaining purified intact fractions of host lipid systems usually requires high quantities of the source sample. For example, the isolation of human plasma lipoproteins requires relatively high quantities of blood. Moreover, it should be provided from a large pool of different blood donors, so that the composition of the lipoproteins is as representative and consistent from one isolation batch to the next as possible. In general, the purification of these host lipid systems is done with complex and long protocols, frequently requiring a second purification step. However, LDs isolation and purification can be relatively simple, if compared to other host lipid systems. Typically, LDs isolation involves stimulation of its production in a particular cell line of interest, after which the cells are lysed in a controlled manner and LDs purified. A number of cell lines have been used for this purpose, including HeLa cells (Kaczocha et al., 2010; Dejgaard and Presley, 2014), hepatocytes (Turró et al., 2006), sebaceous gland cells (Dahlhoff et al., 2015), adipocytes (Martin and Parton, 2008), and baby hamster kidney (BHK) cells (Samsa et al., 2009).

### LDs Isolation

To induce an increased production of LDs within the cell, cell cultures are commonly treated for 24–48 h with oleic acid (Carvalho et al., 2012), oleic acid complexed to defatted bovine serum albumin (Kaczocha et al., 2010) or linoleic acid (Dahlhoff et al., 2015). These are fatty acids that stimulate the fatty acid receptor FFAR4 and enhance the number and size of LDs (Dahlhoff et al., 2015). Following, an analysis of lipid accumulation to evaluate the number and size of LDs can be performed. After the induction, cells have to be washed and resuspended in buffer with a protease inhibitor cocktail. Inhibition of proteases activity is a key step of this protocol; otherwise the results obtained in the subsequent studies can be inaccurate due to changes in the LDs proteome. Following, in the presence of protease inhibitors, cells can be disrupted by nitrogen cavitation using a cell disruption vessel (Samsa et al., 2009), sonication (Martin and Parton, 2008), strokes on ice (Rösch et al., 2017) or shearing with small-bore needles. The method chosen for cell disruption is different in several protocols described in the literature and differs with the type of LDs source (Ding et al., 2013). There is no evident correlation between the method used for cell disruption and the quantity and/or purity of the LDs obtained.

**FIGURE 4:**
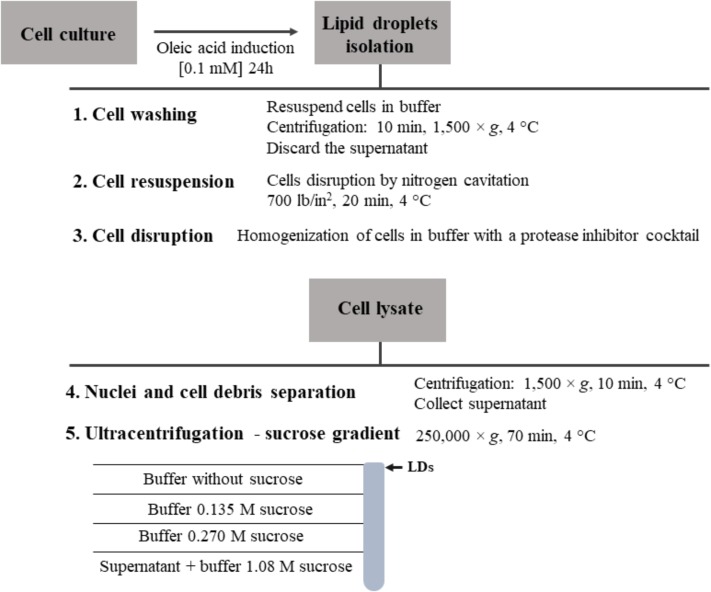
Schematic representation of LDs isolation and purification.

Lipid droplets can be purified from a lysate of cells submitted to centrifugation, since LDs will float in the aqueous gradients. Submitting the cell lysate to a centrifugation at 1,500 × *g* for 10 min is sufficient to remove the nuclei and collect the LDs in the supernatant. With an ultracentrifugation of the supernatant at 250,000 × *g* for 70 min, at 4°C, in a sucrose gradient, it is possible to isolate LDs by collecting fraction from the top to the bottom of the gradient (**Figure 4**) (Carvalho et al., 2012). However, the ultracentrifugation conditions may need to be optimized according to the size of the LDs (Ding et al., 2013). There are other methods to extract LDs, using organic solvents (Matsumoto et al., 2002), but those approaches are less frequently used. Isolated LDs can be tested for the absence of cytosolic contamination, activity of lactate dehydrogenase and the presence of classical LD proteins (Carvalho et al., 2012), as well as via microscopy visualization or other biochemical and biophysical assays (Martin and Parton, 2008).

### LDs Purification Issues

Secondary purification steps are normally required to obtain LDs samples without contaminations. Minimizing contaminants is of special importance when seeking to accurately characterize LDs proteome, a particular difficult step given LDs’ multiple contacts with other intracellular organelles and proteins. LDs-associated proteins are essential for their biogenesis and indispensable for the functions of these organelles. The proteins associated to the surface of LDs vary between cell types (Gao and Goodman, 2015). The first LDs-associated protein identified was perilipin (now termed PLIN1), in 1991 (Greenberg et al., 1991). Since then, several studies have been done to characterize the LDs proteome in different cell lines (Dahlhoff et al., 2015). This was possible with the improvement of different approaches used to characterize proteins, but also due to the improvement of LDs purification protocols. The fractions of LDs collected from the ultracentrifugation gradient may be submitted to an additional washing step, to reduce the contamination of proteins prevenient from other sources (Ding et al., 2013). Several washing steps can be done. However, it is important to keep in mind that very small LDs may be lost with sequential washings. Even with this additional step, it is almost impossible to eliminate the proteins from organelles or membrane structures that are bound to LDs. To disrupt the binding of these proteins to LDs, the pH of the washing buffer can be adjusted to 11.5 (Brasaemle et al., 2004). Although these steps can be very efficient to remove LDs’ contaminating proteins, LDs’ morphology and associated proteins may also be affected. To avoid this, one can perform a quantitative analysis of the proteins in the two fractions separated via the density gradient ultracentrifugation of the cell lysate: the first one corresponding to the proteins in the LDs fraction and the second corresponding to the proteins in the cell pellet containing the remaining cellular components. LDs-associated proteins can be precipitated with trichloroacetic acid and acetone (Boulant et al., 2007) or solubilized in Laemmli buffer (Dahlhoff et al., 2015). Protein fractions can then be separated by SDS-PAGE and analyzed by mass spectrometry. A quantitative comparison of the proteins present in the LDs and pellet fractions can provide accurate information of LD-associated proteins. The criteria defined by Dahlhoff et al. (2015) were that if the amount of a protein is at least the double in the LDs fraction compared to the cell pellet fraction and if this result is reproducible in independent measurements, the protein can be considered as part of the LDs proteome. Using this arbitrary approach, it is possible to identify the LD-associated proteins for a specific type of cells. However, it is important to compare the results obtained with the data already available for the same type and other types of cells. If a protein is identified for the first time as LD-associated, other techniques should be used to confirm the result. For instance, immunofluorescence studies may confirm if the protein presents cytoplasmic localization or if it co-localizes with LDs.

## Methods for Lipid Droplets Characterization

To move forward, it is important to know the arsenal of techniques and approaches through which these crucial organelles can be studied and characterized. LDs have been evaluated concerning their physical chemistry properties such as size, surface charge, zeta potential and molecular weight, among other properties. Additionally, determining their composition in terms of surface proteins is also extremely important (not described here; for more information see: Bersuker et al., 2018; Goodman, 2018; Prévost et al., 2018), not only for a fundamental characterization of LD properties, but also to understand the role of these organelles in pathologies and viral infection. Different techniques and approaches have been used to better understand LD-mediated processes and their role in *Flaviviridae* infection. Recent methodological improvements provided new approaches to study these organelles and to identify specific viral and host factors involved in key steps of the virus life cycle. The information gathered by these methods may be combined to develop drug strategies against important pathogens of the *Flaviviridae* family. Here, we present different approaches to characterize LDs within the cell environment or isolated from cell cultures. In particular, we review light scattering, zeta potential and microscopy techniques, including atomic force microcopy-based force spectroscopy, in the context of the use of such techniques to study LDs role in the viral life cycle.

### Light Scattering

Dynamic light scattering (DLS) spectroscopy is a technique commonly used to determine the size distribution profile of particles in suspension (Domingues et al., 2008). DLS is used to measure the hydrodynamic diameter and size distribution of molecules or supramolecular aggregates, based on the light scattering intensity fluctuation on a small volume, in a time-scale of microseconds, due to the Brownian motion of the particles (Domingues et al., 2008; Stetefeld et al., 2016). The scattered light is collected and measured at a given angle by a sensitive detector. Size determinations can be performed through the measurement of the scattering light intensity fluctuations as a function of time, since the diffusion rate of particles is determined by their size. To calculate the correlation kinetics, which depends on the intensity-weighted diffusion coefficient (*D*), different methods can be employed, such as CONTIN (Provencher, 1982) or Cumulants (Frisken, 2001). With the Stokes–Einstein equation, it is possible determine the hydrodynamic diameter (*D_H_*) from the diffusion coefficient (Berne and Pecora, 1990):

$$D_{H}=\frac{\kappa T}{3\pi \eta D}$$

where η is the dispersant viscosity, *κ* the Boltzmann constant and *T* the absolute temperature. It should be noticed that the exponentially decaying curve goes to zero at time when the particle in movement exceeds the wavelength of the laser light (Uskoković, 2012). The scattering intensity distribution function of *D_H_*, [*I*(*D_H_*)], is obtained, and can be converted to *n*(*D_H_*), the particle number distribution function of *D_H_* through the Mie theory (Santos and Castanho, 1996; Faustino et al., 2014). The scattering intensity of a particle is proportional to the sixth power of its *D_H_* (Rayleigh’s approximation); thus, the conversion can be done by the following transformation (Santos and Castanho, 1996; Faustino et al., 2014):

$$n(D_{H})\approx \frac{I(D_{H})}{{D_{H}}^{6}}$$

*n*(*D_H_*) expresses how much a particle of a certain diameter scatters light.

DLS experiments can be performed in a Malvern Zetasizer Nano ZS equipped with a He–Ne laser, λ = 632.8 nm, with a backscattering detection at 173° (Faustino et al., 2014). The size of particles in suspension can be determined in terms of *D_H_*, analyzing the normalized intensity autocorrelation functions.

Dynamic light scattering can provide quantitative information on particle size and shape, with relatively fast measurements (Faustino et al., 2014). However, for heterogeneous and highly polydisperse systems, the results can be inaccurate (Vezočnik et al., 2015). If the LDs sample in study is highly heterogeneous, which may naturally occur, the light scattered from larger LDs may obscure the light scattered from the smaller ones. In this case, the determined size distribution probably will not correspond to the real situation or the heterogeneity in size will not allow a proper estimation of size.

Asymmetric-flow field-flow fractionation technique (AF4) coupled to a multi-angle light-scattering (MALS) enables the separation of particle accordingly with their size and the determination of size distribution, total number, and number density distribution of particles (Vezočnik et al., 2015). Recently, Sitar et al. (2017) introduced an AF4 to a MALS detector with an embedded DLS module to study the size characteristics and shape of artificial LDs. Flow DLS experiments, with a flow rate of 0.2 mL/min, gave accurate hydrodynamic radius (*R_H_*) values (Sitar et al., 2017). Although, with increasing flow rates at the DLS detector, the accuracy of *R_H_* determination is lower (Sitar et al., 2017).

More recently, a new method for monitoring LDs size based on light scattering was proposed, nanoparticle tracking analysis (NTA) (Muratore et al., 2018). The size distributions of LDs could be measured using a Nanosight LM-10 Nanoparticle Tracking Analyzer, equipped with a 405 nm laser and a high sensitivity camera. The light scattered revealed the temporal positions of individual LDs, which are recorded with a camera. To calculate the *R_H_* of LDs, the motion of each LD is tracked individually from the frames of the captured videos (Muratore et al., 2018).

The choice of the type of light scattering measurements should be done based on the sample characteristics. To determine the *D_H_* or *R_H_* of highly homogeneous LDs samples without high propensity to aggregate, DLS measurements assure accurate and reliable results. Otherwise, other methods should be chosen. NTA was already applied to measure the size of LDs isolated from mouse liver. Moreover, in the study performed with this technique, it was possible to analyze the size distribution of LDs from adult and geriatric mice (Muratore et al., 2018). This method can now be applied to determine LDs size isolated from different tissues or cells. NTA may be a powerful technique to compare LDs size from non-infected and infected cells. The possible achievements may allow understanding the effect of different viruses on LDs.

### Zeta Potential

Zeta potential (ζ-potential) measurements are based on the concept that charged particles in suspension attract to their surface ions with opposite charge, to which they can be strongly bound. These surface-bound ions form a layer, the Stern layer (Uskoković, 2012). Beyond the Stern layer, another layer is formed, where ions diffuse more freely. When the particle moves in the solution, the ions strongly attached to their surface move with it, whereas the ions in the diffuse boundary do not move with the particle. The potential that exists at this boundary is defined as the ζ-potential (Domingues et al., 2008). The ζ-potential is calculated through the electrophoretic mobility of the particles in solution, on an electric field, to the electrode of opposite charge (Kirby and Hasselbrink, 2004). The viscous forces oppose the movement of the particles in suspension until reaching the equilibrium and, therefore, a constant velocity. The electrophoretic mobility can be calculated based on phase analysis light scattering. The ζ-potential of the particles can be calculated using the Henry’s relation (Domingues et al., 2008):

$$\zeta =\frac{3\eta u}{2\epsilon f\left(ka\right)}$$

where ζ is the ζ-potential, *u* the electrophoretic mobility, η the viscosity of the solvent, ε its dielectric constant and *f*(*ka*) is the Henry’s function.

**FIGURE 5:**
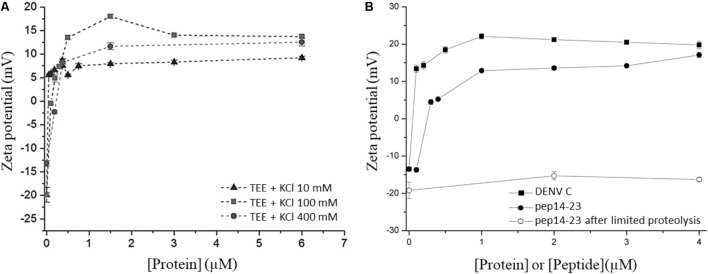
ζ-potential of LDs in different conditions. **(A)** LDs ζ-potential values determined in the absence and in the presence of distinct DENV C concentrations, at different KCl concentrations (adapted from Carvalho et al., 2012). **(B)** LDs ζ-potential values determined before (filled symbols) or after limited proteolysis (empty symbol), in the presence of DENV C (squares) or pep14-23 (circles) (adapted from Martins et al., 2012).

ζ-potential measurements were performed to study the interaction of DENV C protein with LDs, isolated from human hepatocellular liver carcinoma cells (HepG2) (Carvalho et al., 2012). To understand the LDs surface charges in the interaction with the DENV C, the ζ-potential of LDs was determined in the absence and presence of different concentrations of this viral protein (**Figure 5A**). LDs presented a ζ-potential of -19 mV in buffer with 100 mM KCl. The addition of DENV C led the LDs ζ-potential to increase to 13.7 mV at the highest C protein concentration tested (6 μM) (Carvalho et al., 2012). The C protein forms a homodimer in solution and is proposed that one of the faces of the protein is hydrophobic and the opposite face is positively charged (Samsa et al., 2009; Martins et al., 2012). Such an approach showed that the C protein interacts with LDs and that it likely exposes its positively charged α4-α4′ region to the aqueous environment. Measuring the ζ-potential of LDs in the presence of increasing concentrations of C protein showed that a saturation point was reached (Carvalho et al., 2012; Martins et al., 2012). Thus, ζ-potential measurements allowed the estimation of the concentration of a protein ligand needed to saturate the binding sites at the LDs surface. In addition, the variation of ζ-potential (Δζ), induced by the interaction of a protein with LDs, can be calculated by subtracting the measured to the initial values. Then, the variation in the ζ-potential of LDs can be represented as a function of C protein concentration, fitting the experimental data with the following equation:

$$\Delta \zeta =\frac{{\Delta \zeta }_{\max}\left[\text{C protein}\right]}{C_{1/2}+[\text{C protein}]}$$

where Δζ_max_ is the maximum amplitude of variation of the ζ-potential induced by the interaction with C protein and *C*_1/2_ is the C protein concentration at Δζ_max_/2. The values of Δζ, Δζ_max_, and *C*_1/2_ can be used to understand the interaction of proteins or peptides with LDs in different conditions (**Table 1**). For example, changing the ionic strength or the ions of the buffer it is possible to understand if the interaction of the protein with LDs depends on specific ions or is only affected by ionic strength. Another possible approach to study LDs interaction is submitting LDs to limited proteolysis. As described above, LDs have several proteins on their surface that can mediate the interactions with these organelles. By submitting LDs to a limited proteolysis, the proteins at the LDs surface will be removed, after which the ζ-potential of these LDs can be measured. These LDs without (part of) their surface proteins can then also be allowed to interact with a protein of interest and the ζ-potential variation can then be calculated. This enables the determination if a protein of interest interacts with LDs-surface proteins or lipids, by comparing their ζ-potential with the ζ-potential value obtained for LDs not submitted to proteolysis in the presence of the same protein. This approach allowed us to find that DENV C binding to LDs is dependent of surface proteins of LDs, as well as potassium ions (Carvalho et al., 2012). LDs ζ-potential in the presence of DENV C in buffers with different potassium concentrations (10 mM, 100 mM or 400 mM KCl) showed a higher ζ_max_ variation (Δζ_max_) in the presence of 100 mM of KCl. This approach can be used to study other types of interaction, such as LDs interaction with possible inhibitor peptides (Martins et al., 2012). Measuring the ζ-potential of LDs in the presence of an inhibitor peptide of DENV C interaction with LDs, pep14-23, a variation of LDs’ ζ-potential from -20 mV (in the absence of the peptide) to positive values (in the presence of the peptide) was observed (**Figure 5B**). It was found that pep14-23 binds to LDs and may inhibit their interaction with DENV C (Martins et al., 2012). The approach used to characterize DENV C–LDs interaction in terms of ionic strength and LDs surface proteins as targets can now be applied to other viruses. WNV and ZIKV, which have similar C proteins conserved in terms of sequence and structure, are obvious candidates.

**Table 1:** Parameters obtained by ζ-potential analysis of LDs under different conditions.

Condition		Mean *C*_1/2_ ± SE (nM)	Mean Δζ_max_ ± SE (mV)
KCl (mM)	10	5.9 ± 2.2	27.9 ± 0.4
	100	87.7 ± 17.6	34.4 ± 1.3
	400	188.4 ± 68.8	27.6 ± 2.3


*The values of *C*_1/2_ and Δζ_max_ were obtained through the fitting of ζ-potential experimental data with equation 4. Adapted from Carvalho et al. (2012).*

### Optical Microscopies

Microscopy studies are frequently used to identify the intracellular localization of LDs. Moreover, with optical microscopies it is possible to extract information of LDs morphology. LDs can be identified using a fluorescent staining for the neutral lipid core or without staining, using the intrinsic properties of the lipid core in bright field, differential interference contrast (DIC) or phase contrast microscopy (Martin and Parton, 2008). Several dyes considered to be specific for LDs have been used to detect intracellular LDs, such as BODIPY, one of the most commonly used (Peramuna and Summers, 2014; Dahlhoff et al., 2015), LipidTox Deep Red (Kozusko et al., 2015), Oil Red O (Nevo-Yassaf et al., 2017), and Nile red (Cohen et al., 2017). However, fluorescent dyes present disadvantages that can lead to inaccurate results. If the analysis involves the detection of multiple fluorophores, which may have broad and partially superimposing excitation and emission spectra, the data can be hard to analyze. Besides, many fluorophores are unstable and tend to photobleach rapidly. Most importantly, fluorophores that are hydrophobic may affect LDs structure (Martin and Parton, 2008; Appelqvist et al., 2017). Recently, a benzothiadiazole dye derivative with solvatochromic properties (LD-BTD1) revealed to be very efficient, exhibiting a non-toxic profile and a high signal-to-noise ratio in cells (Appelqvist et al., 2017). To study LDs dynamics in cells by confocal microscopy, the LD-BTD1 dye should be used. This dye is specific for intracellular LDs and exhibits strong solvatochromic behavior in solvents with increasing polarity (Appelqvist et al., 2017). The properties of this dye enhance the contrast between the stained LDs and the cytosol, reducing the background. Furthermore, LD-BTD1 does not have a pendant oleic acid tail, but can be used to study dynamic changes in LDs biology.

To acquire LDs images of fixed cells, it is necessary to perform a fixation step followed by a labeling step. There are several protocols described in the literature for LDs fixation and permeabilization. However, there are evidences that most of them promote alterations in the structure of LDs (Fukumoto and Fujimoto, 2002; DiDonato and Brasaemle, 2003; Ohsaki et al., 2005). To preserve LDs’ structure for light microscopy studies, the fixation should be done with paraformaldehyde in phosphate buffered saline (PBS) and the permeabilization with saponin (Martin and Parton, 2008). Alternatively to cell fixation, an isolated fraction of purified LDs can be directly applied to glass coverslips pretreated with poly L-lysine (Martin and Parton, 2008). After LD or cell fixation and permeabilization, LDs can be stained using a dye specific for neutral lipids. Other possibility is the immunolabeling of LDs. In this case, cells or LDs are incubated with a primary antibody in a blocking solution, washed and then incubated with a secondary fluorescently conjugated antibody in the same blocking solution. These two types of LD labeling can be done in parallel by adding simultaneously the dye and the secondary antibody.

Confocal microscopy has been extensively used to study LDs in terms of structure, accumulation and dynamics. With this type of microscopy studies, LDs can be imaged in three dimensions (two dimension stacks), allowing the determination of LDs volume. For example, LDs composition and accumulation was studied by staining LDs with BODIPY 505/515 (Peramuna and Summers, 2014). To quantify LDs formation, the percentage area of LDs per cell was measured from digital images, obtained with a Leica LAS AF confocal laser microscope, using the software ImageJ^1^. The area of the lipids stained by BODIPY can be measured by tracing the outline of the LDs using the freehand selection function and measuring the pixel area within the chosen area. Moreover, it is possible to determine the percentage area occupied by LDs per cell, by dividing the pixel area of the lipids by the pixel area of the cell and multiplying by 100 (Peramuna and Summers, 2014).

**FIGURE 6:**
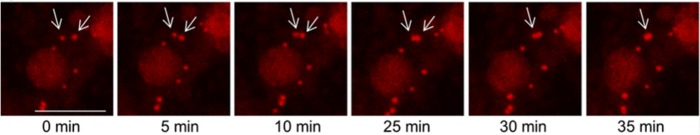
Representative fusion event of two lipid droplets. Fusion of two lipid droplets observed in mammary epithelial cells (MEC) treated with 100 μM free palmitic acid + 10 μM 3-deazaadenosine. LDs were stained with Nile red and MEC were imaged on a time-lapse system. Scale bar: 5 μm [reprinted by permission from Springer Nature: Springer United States (Cohen et al., 2017), Copyright (2017)].

In fact, with fluorescence and confocal microscopy, it has been possible to understand the LDs regulation mechanism. Recently, a regulatory mechanism of LDs size was identified, which is independent of cell TAG content and may be regulated by a fusion pathway (Cohen et al., 2017). Mammary epithelial cells were grown on glass cover slips, stained with 4’,6-diamidino-2-phenylindole (DAPI), and visualized with an Olympus BX40 fluorescence microscope equipped with an Olympus DP73 digital camera using CellSens Entry software. LDs diameter was measured using ImageJ. Authors considered cells with at least one LD larger than 2.5 μm as “large LDs” and cells with all LDs below or equal to this diameter threshold as “small LDs.” To analyze LDs fusion, they performed live cell imaging with a Leica TCS SP8 confocal microscope. LDs were detected by adding Nile red directly to the treatment medium. Pictures of confocal planes through the cells were taken, using Leica LAS AF software. Then, a 3D reconstruction of the relevant frames was conducted and visualized to validate LDs fusion (**Figure 6**) (Cohen et al., 2017).

**FIGURE 7:**
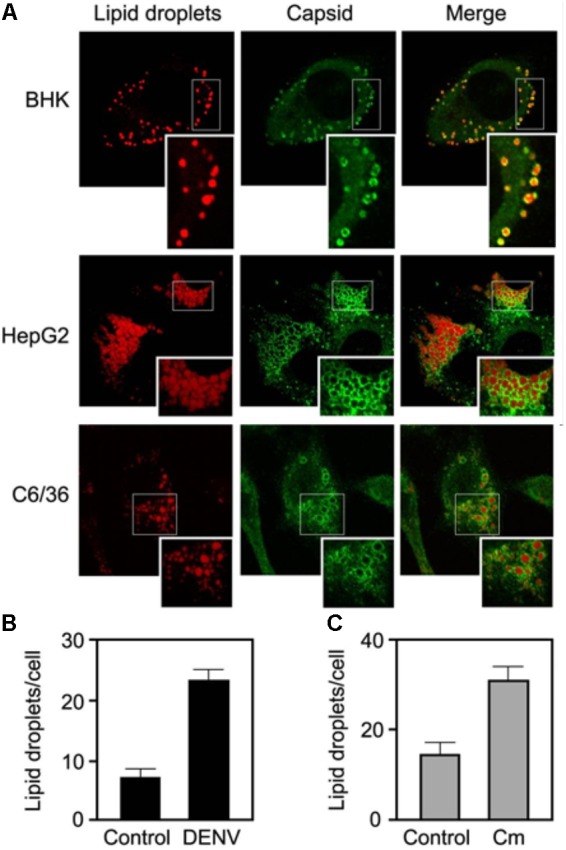
DENV infected cells accumulate the C protein around LDs. **(A)** DENV C is targeted to LDs in BHK, HepG2, and C6/36 cells infected with DENV. **(B)** DENV infection increases the number of LDs. **(C)** DENV C expression increases the number of LDs (adapted from Samsa et al., 2009).

By confocal microscopy it was also shown that HCV core protein localizes at the surface of LDs. For that, LDs were stained with Oil Red O and HCV core protein was detected with a primary antibody and an anti-mouse secondary antibody conjugated with FITC (McLauchlan and Hope, 2000). The same approach used to detect the localization of HCV core protein at LDs surface was used to detect mature DENV on the surface of LDs in infected cells (**Figure 7A**) (Samsa et al., 2009). LDs were stained with BODIPY 493/503 and the C protein was detected with a rabbit polyclonal antibody against DENV C and a Cy3-conjugated goat anti-rabbit IgG. Visualizing the LDs in infected and control cells shows a threefold increase on the number of LDs in DENV-infected cells (Samsa et al., 2009) (**Figures 7B,C**). With this approach, it was possible to test the effect of a FAS inhibitor (C75) on LDs amount in DENV-infected and uninfected cells, showing that it reduces LDs number. Moreover, C75 also induced an 100- to 1,000-fold inhibition of DENV replication, as well as a reduction of viral particle production, probably due to the decreased amount of LDs. Such approaches are thus quite powerful and informative.

Microscopy studies were also performed to study DENV pathogenesis. It was reported that patients with DHF have elevated plasma concentrations of macrophage migration inhibitory factor (MIF). Leukocytes from these patients and healthy donors were stained with osmium tetroxide or BODIPY 493/503, and analyzed by light microscopy or phase-contrast and fluorescence microscopy, respectively, to detect the presence of LDs. Cells from patients with DHF revealed a threefold increase in LD accumulation compared with cells from healthy donors. Moreover, LDs from patients with DHF showed higher amounts of MIF. These results suggest that LDs quantification can be an indicator of the disease severity (Assunção-Miranda et al., 2010).

Another confocal microcopy study was performed to understand the role of autophagy in DENV infection (Heaton and Randall, 2010). A kinetic analysis of autophagosome association with LDs showed a maximal association 24 h after DENV infection. LDs diameter was measured using ImageJ and it was observed that the diameter of LDs in DENV-infected cells was approximately 35% decreased as compared to mock-infected cells. LDs volume was calculated using the average diameters obtained by microscopy and the equation of the volume of a sphere. A reduction of LDs volume of approximately 70% was calculated for the LDs in infected cells. Authors concluded that autophagy is required for DENV replication, providing a mechanism by which viruses can alter cellular lipid metabolism to promote their replication (Heaton and Randall, 2010). Therefore, it is possible that viruses induce LDs accumulation for an efficient viral replication. After that, LDs number decreases due to an autophagy-dependent processing of LDs to release free fatty acids.

Real-time visualization of LDs in cultured cells can provide crucial information to understand their role in pathogenesis. A comparison of LDs biogenesis and dynamics in infected and non-infected cells allows the identification of LDs’ processes occurring due to viral infection (Nevo-Yassaf et al., 2017). LDs biogenesis was also observed via these approaches, showing that LDs may be formed from peripheral TAG accumulations on ER membranes. Moreover, it was demonstrated that HCV NS5A interacts with loci at ER membranes where LDs may be formed (Nevo-Yassaf et al., 2017).

### Electron Microscopy

**FIGURE 8:**
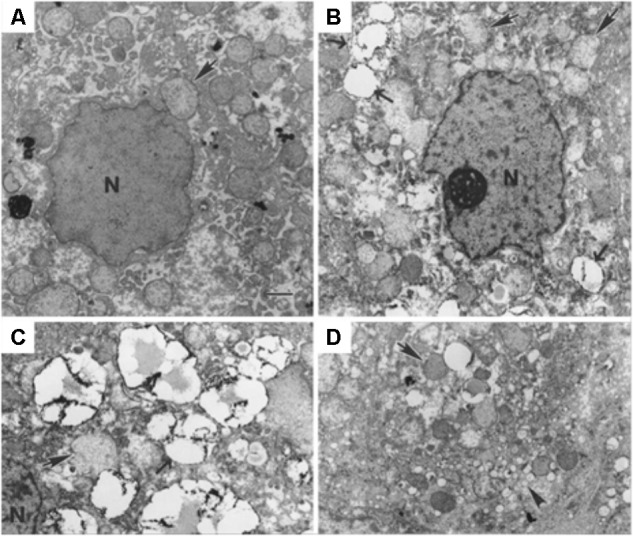
Transmission electron microscopy of cells infected with viruses, showing LDs morphological changes. **(A)** Tamarin liver tissue uninfected and **(B–D)** infected with GB virus B, an unclassified flavivirus similar to HCV. Lipid droplets (pointed by →, and with electron-dense surface deposits), mitochondria (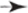) and the nucleus (N) can be identified. The scale bar corresponds to 1 μm. Most hepatocytes of the infected animal contain multiple small (*ca.* 2 μm diameter) LDs. They may be indicative of ongoing viral replication [reprinted from Martin et al. (2003). Copyright (2003) National Academy of Sciences, United States].

Alternatively, LDs can be identified by electron microscopy. In fact, this technique presents a higher resolution, allowing a better characterization of LDs in terms of structure, formation and interaction with other organelles. Electron microscopy imaging allowed to identify LD-associated proteins and whether these proteins were associated with LDs surface, core or with the LD-associated membranes (Martin and Parton, 2008). Transmission electron microscopy (TEM) images were used to show that GBV-B, a hepatotropic virus and a close relative of HCV, leads to LDs accumulation in hepatocytes, which may even migrate from the cytoplasm to the nucleus (**Figure 8**). LDs number and arrangement, observed via TEM in liver biopsies, were considered as indicative of latent infection and may contribute to a differential diagnosis (Martin et al., 2003).

As described in the previous section, the use of dyes with lower selectivity may lead to inaccurate results, due to their incorporation into other cellular structures and/or to variations of their cellular distribution. Studying LDs accumulation and structure in infected cells or tissues with electron microscopy can overcome the problems related with the use of lipophilic dyes. It could be of great interest to study LDs localization in *Flavivirus*-infected cells, as well as their size and structure. Moreover, complementing with results obtained with different techniques, it should be possible to evaluate which viruses use an autophagy-dependent processing of LDs, by analyzing the number and size of LDs inside a cell.

### Stimulated Raman Scattering Microscopy

**FIGURE 9:**
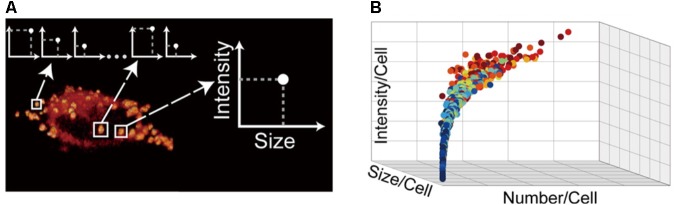
Stimulated Raman scattering image processing. **(A)** The intensity and size of each LD can be measured. **(B)** For each cell, cultured with different concentrations of oleic acid, the total size, intensity, and number of LDs can be quantified. The relation of these LD morphology parameters among single cells can be inferred under various culture conditions. A dot-plot shows a possible relation among those parameters. The different colors of the dots represent different culture conditions [adapted with permission from Cao et al. (2016). Copyright (2016) American Chemical Society].

Usually, LDs characterization is done with LD samples isolated from cell cultures. However, it is extremely important to characterize LDs from a single cell. Studies of the heterogeneity of LDs in different cells can be highly significant to understand LDs function. Microscopy imaging has been used to collect data from single cells, particularly from LDs localization, morphology, number and size. However, the efficiency of fluorescent labeling can be lower, unspecific binding may occur, and fluorescent molecules may alter LDs’ structural properties. Recently, to overcome these problems, LDs quantification was done by stimulated Raman scattering (SRS) microscopy studies. SRS is a powerful tool for imaging without labeling. The sample is excited by two lasers and, when the variation of the two frequencies is equal to a particular Raman active molecular vibration of the sample, SRS signals is generated due to the non-linear interaction between the photons and the molecules (Lu et al., 2012). Cao et al. (2016) combined microfluidic technology and SRS microscopy to quantitatively characterize LDs with a single-LD resolution at the single-cell level. Cells are cultured and treated in a microfluidic device and then imaged by SRS microscopy, followed by a quantitative image analysis. Assuming that the intensity of the SRS signal is proportional to the number of specific chemical bonds in the detection foci, it is possible to identify the LDs. However, other organelles with membrane enriched structures may also be probed by SRS on the lipids band and generate irregular background patterns. Thus, background removal is crucial in SRS image processing (Lu et al., 2012; Cao et al., 2016). Once the background from other organelles is removed, it is possible to calculate LDs size by counting the number of pixels per droplet and then mapping each pixel’s intensity back to the mask area and adding them across the image stack (**Figure 9**). With this technique, it is possible to construct distributions of the morphological parameters of LDs in the cellular population and perform a statistical single-cell quantification of them (Cao et al., 2016).

### Atomic Force Microscopy

Atomic force microscopy (AFM) is one of the techniques from the group of scanning probe microscopies (Carvalho and Santos, 2012). This technique is based on the scanning of a sample surface by a probe, evaluating the interaction between a sharp tip and the surface of the sample with precise spatial location (Santos and Castanho, 2004). This interaction force between tip and sample depends essentially on three parameters: the sample nature, the probe tip and the distance between them. To scan the surface, the tip can essentially do two different movements: drag across the surface or vibrate as it moves along it (Whited and Park, 2014). Typically, AFM is used for the construction of surface images. A tip attached to a flexible cantilever scans or taps the surface, while a laser beam reflected on the back of the cantilever is detected with a position-sensitive photodiode. Initially, the laser is pointed at the center of the photodiode, usually composed of four quadrants. When the sample is scanned, any small deflection on the cantilever will change the position of the reflected laser (lateral and vertical deflections of the tip are distinguishable). These deflections of the tip are processed by the electronic system and the sample surface topography is determined (Vahabi et al., 2013). Furthermore, with AFM it is possible to image in air and in liquid, as well as in non-conductive and conductive surfaces.

**FIGURE 10:**
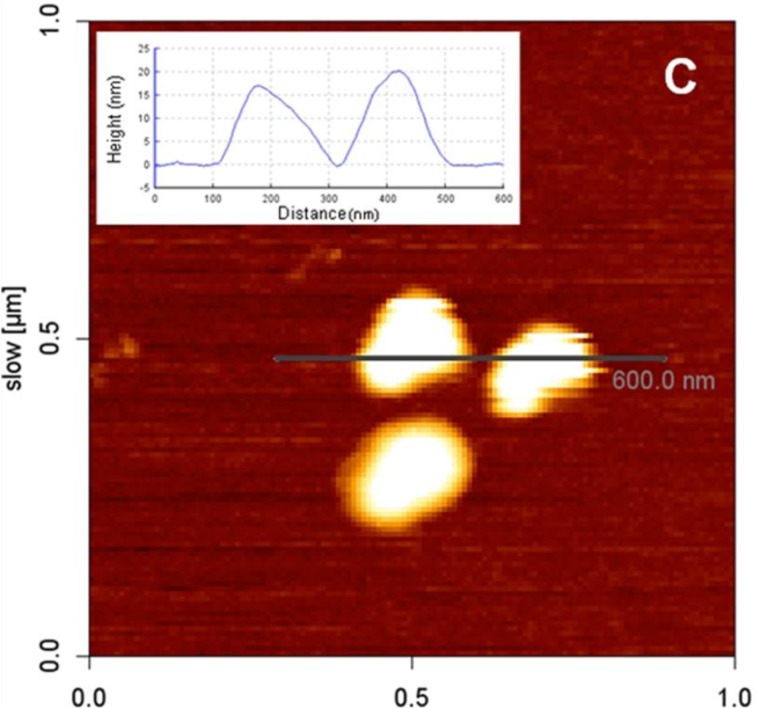
Atomic force microscopy image of LDs isolated from HepG2 cells. LDs were deposited onto freshly cleaved muscovite mica. The inset shows a cross-section profile of two LDs with a diameter of approximately 200 nm and height of 15–20 nm (adapted from Carvalho et al., 2012).

To acquire images of LDs with AFM, a purified fraction of LDs should be used. LDs suspension can be placed onto thin freshly cleaved muscovite mica (Carvalho et al., 2012; Martins et al., 2012). This will allow LDs to adhere without affecting their structure. Following, a washing step with buffer has to be done to remove the non-adherent LDs. Deposited LDs can be scanned with the AFM tip and LDs diameter and height can be measured (**Figure 10**) (Carvalho et al., 2012).

### AFM-Based Force Spectroscopy

In addition to the scanning of a sample surface, AFM can be used to measure the interaction forces between molecules. Taking advantage of its piconewton sensitivity, one can quantify interactions between a functionalized tip (described ahead) and a specific spot of the sample. AFM can be a powerful tool for the study of molecular interactions (Carvalho et al., 2013; Guedes et al., 2016). AFM-based force spectroscopy is a technique that allows the measurement of inter- and intramolecular interaction forces required to separate the tip from the sample, enabling the detection of specific interaction forces at the single molecule level. For this type of studies the tips are functionalized with a probe molecule to recognize a specific target on the sample surface (Carvalho and Santos, 2012; Vahabi et al., 2013; Whited and Park, 2014). In AFM-based force spectroscopy measurements at a single-molecule level, the tip is brought into contact with the sample surface and then retracted. For this, the cantilever moves first toward the surface and then in the opposite direction, upward (in the *z-*axis). The cantilever deflection of the vertical displacement of the piezoscanner can be recorded as cantilever deflection vs. scanner displacement in terms of height. When the tip starts to approach the sample, there are no changes on this deflection-distance plot. When the tip touches the sample, the force applied increases, leading to the deflection of the cantilever. When a given deflection (or force) value is reached, the tip starts to retract, and a curve with the same appearance of the approach is generated. If a molecule attached to the tip, or simply the tip, adheres to the sample, a binding event occurs and the retraction curve presents a different shape. This deflection-distance information can be converted into a force-distance curve using the Hookes’s law of elasticity (Carvalho and Santos, 2012):

F=−kΔx

where *F* is the force, *k* the spring constant of the cantilever and Δ*x* the length of the deflection of the cantilever. The tip, sample and medium composition influence the curve obtained. The covalent coupling of biomolecules to the AFM tip is another point to take into account for the force measurement. It is essential that the molecules can be removed from the surface during the retraction curve, but not from the tip (Carvalho and Santos, 2012).

Atomic force microscopy-based force spectroscopy studies have been performed to measure the interactions of DENV C (covalently attached to the AFM tip) with LDs (lightly adsorbed to a mica surface, as described above), in buffers of different composition (Carvalho et al., 2012; Martins et al., 2012). The use of glutaraldehyde as a flexible cross-linker to couple the C protein to the tip allows the protein to diffuse freely, with its binding sites available to establish bonds with its ligands (Willemsen et al., 2000). By tapping with this functionalized tip on the surface of the deposited LDs, the detection of binding events and the force necessary to break the interaction can be determined at the single-molecule level. The number of proteins bound to the functionalized tip is unknown; however, this information is not required for force measurements, since it is possible to identify single-molecule binding events from the shape of the retraction curve. To have the data statistically validated, the acquisition of hundreds or thousands of curves is necessary, as well as the use of more than one AFM tip for each condition (Carvalho and Santos, 2012; Carvalho et al., 2012). With this information, histograms of distribution of rupture forces or distances can be generated, and the frequency of (un)binding events calculated from the number of curves with (un)binding events over the total number of curves obtained. The histograms distribution may be analyzed with a Gaussian model to extract the average force necessary to break the bond between the protein attached to the AFM tip and the LD. If more than one protein attached to the AFM tip interacts with LDs, multiple peaks of the histograms distribution will be fitted with the Gaussian model (Carvalho et al., 2010; Carvalho and Santos, 2012). However, if the protein attached to the AFM tip does not bind specifically to the LD, only a peak corresponding to unspecific interactions will appear in the histogram, at low force values.

**FIGURE 11:**
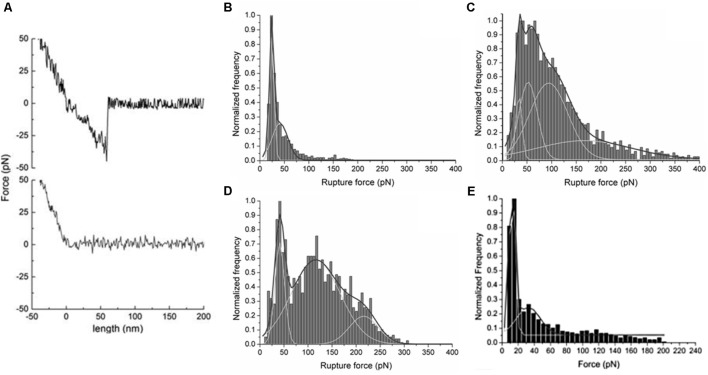
AFM-based force spectroscopy results for DENV C interaction with LDs. **(A)** Typical force-extension curves of a control experiment with an unmodified silicon nitride AFM tip interacting with the mica surface (gray line), where adhesion forces were undetectable, and of a DENV C-functionalized tip interacting with a LD isolated from HepG2 cells (black line). Force rupture histograms of DENV C–LDs binding in buffer with **(B)** 10 mM KCl, **(C)** 100 mM KCl, **(D)** 400 mM KCl (adapted from Carvalho et al., 2012) or **(E)** 100 mM KCl plus 100 μM pep14-23 (adapted from Martins et al., 2012).

Using AFM-based force spectroscopy, it is possible to evaluate the types of force involved in the interactions with LDs, performing force measurements under different buffer conditions (buffers with different ions or different concentrations) (Carvalho et al., 2012) or with LDs submitted to a limited proteolysis (see “Zeta Potential” section) (Martins et al., 2012). The rupture forces and the percentage of (un)binding events of DENV C–LDs interaction in the presence of different ions, as well as in different ions concentration, gave details about this interaction (**Figures 11A–D**). Only in the presence of physiological high intracellular potassium concentrations were observed multiple peaks on force rupture histograms and the highest percentage of binding events, demonstrating that DENV C–LD interaction is specific (Carvalho et al., 2012). Although this technique allows the determination of specific interactions as well as their binding force, it is complex and time-consuming. However, now that it is well established, it can be performed to study the interaction of other viral proteins with LDs. Moreover, force spectroscopy measurements can be performed to identify specific proteins of the LDs surface responsible for the interaction. For this, the adhered LDs are pre-incubated with antibodies against specific proteins of the LDs surface (Carvalho et al., 2012). Using this approach, AFM-based force spectroscopy measurements showed that DENV C mainly interacts with PLIN3 at the surface of LDs (Carvalho et al., 2012). The results obtained suggest PLIN3 as the main target for DENV C binding. The same experiment can be performed with antibodies against different proteins at the LDs surface, and in the presence of other viral proteins. Comparing the force and percentage of binding events obtained, it may be possible to identify the LD proteins involved in virus replication. Once they have been identified, it could be important to understand if they are present at the surface of other lipid systems. AFM-based force spectroscopy provides accurate data on the force and probability of interactions, at the single-molecule level. Therefore, it is an excellent technique to test the effectiveness of possible inhibitors of crucial interactions of viral proteins with LDs. The peptide pep14-23 was tested by this approach, demonstrating its potential application as inhibitor of DENV C–LDs interaction (**Figure 11E**) and, therefore, of DENV replication (Martins et al., 2012). As soon as other viral proteins involved in specific interactions with LDs-surface proteins have been identified, pep14-23 (and other peptides) can be tested as a potential broad-spectrum inhibitor.

## Conclusion

Lipid droplets are essential organelles, involved on the maintaining of the cellular homeostasis and playing an important role in cellular energy storage and lipid metabolism (Bersuker and Olzmann, 2017). Viruses have the ability to hijack the intracellular membrane machinery for viral replication. Several viruses of the *Flaviviridae* family have been associated to the deregulation of the lipid metabolism. Important human pathogens, such as DENV, HCV, WNV, and ZIKV, are associated, respectively, with hemorrhagic fever (Bhatt et al., 2013), steatosis (McLauchlan, 2009), neurologic illness (Rossi et al., 2010), and microcephaly (Calvet et al., 2016). Despite the knowledge gathered by studies conducted in the last years, there are no effective drugs available against these viruses. The important role of LDs in flaviviruses life cycle makes them a possible target for the development of new therapeutics. A better characterization of LDs morphology, proteome and “interactome” may provide crucial information to understand LDs as fundamental organelles in viral replication. Moreover, the identification and characterization of viral factors, as structural and non-structural viral proteins, as well as their interaction with specific LD factors may provide the information needed to develop effective treatments. Different techniques may be used in further studies, depending of the central question in analysis. To characterize LDs size, techniques based on light scattering such as AF4 and NTA, may provide fast and accurate results. However, LDs have to be isolated from cell cultures. Microscopy studies allow the characterization of LDs in cell cultures or tissues, in terms of size, localization, accumulation and dynamic. Comparative studies of non-infected and infected cell cultures may reveal important details to understand the LDs role in the viral life cycle. Moreover, live cell imaging and analysis of LDs biogenesis may provide crucial information to understand their role in pathogenesis. Although confocal microscopy is one of the most used techniques, it requires the use of fluorescent dyes. SRS can also be used to extract the same information from a single cell and without using dyes. Electron microscopy can be used to characterize LDs size, formation and interaction with other organelles. Moreover, this technique presents a higher resolution. Furthermore, it is known that viral proteins play an important role in several steps of the viral life cycle, namely in viral assembly and encapsidation. Despite all the knowledge on viral proteins interaction with LDs, much more information can be gathered via AFM-based force spectroscopy. Therefore, taking all of the above into consideration, a comprehensive understanding of LDs role in viral infection is fundamental to develop strategies to inhibit viral replication.

## Author Contributions

All authors listed have made a substantial, direct and intellectual contribution to the work, and approved it for publication.

## Conflict of Interest Statement

The authors declare that the research was conducted in the absence of any commercial or financial relationships that could be construed as a potential conflict of interest.

## Footnotes

1 http://imagej.nih.gov
